# A Good Night’s Sleep: Learning About Sleep From Autistic Adolescents’ Personal Accounts

**DOI:** 10.3389/fpsyg.2020.583868

**Published:** 2020-12-22

**Authors:** Georgia Pavlopoulou

**Affiliations:** ^1^Department of Psychology and Human Development, Institute of Education, University College London, London, United Kingdom; ^2^Anna Freud National Centre for Children and Families, London, United Kingdom

**Keywords:** sleep, autism (ASD), phenomenology and care ethics, adolescence, mental health – related quality of life, autistic wellbeing

## Abstract

**Background:**

Sleep is a strong predictor of quality of life and has been related to cognitive and behavioral functioning. However, research has shown that most autistic people experience sleep problems throughout their life. The most common sleep problems include sleep onset delay, frequent night-time wakings and shorter total sleep time. Despite the importance of sleep on many domains, it is still unclear from first-hand accounts what helps autistic people to sleep. The purpose of this study is to explore together with autistic adolescents their sleep-related practices before bedtime and during the day which contribute to a good night’s sleep.

**Methods:**

Fifty-four autistic adolescents collaborated with an academic researcher in a novel adapted photo-elicitation methodology, rooted in a Lifeworld framework. The adolescents were invited to collect and analyze their data. The data were also presented in a community knowledge exchange event.

**Results:**

Several self-reported practices that facilitate better nocturnal sleep were identified. Those were organized into two thematics: Evening/bedtime factors and Day time factors. These included practices such as personalized sensory and relaxation tools before bed and during night-time, engaging in a range of physical activities during daytime and accommodating personal time to engage with highly preferred and intense focus activities and hobbies. It also included spending time in predictable and fun ways with family members before bedtime.

**Conclusion:**

This is the first time that a study uses a novel methodological approach based on personal accounts elicited by photos rooted in a Lifeworld framework to describe personal sleep-related practices before bedtime and during the day to identify a “good night of sleep” in autistic adolescents. The outcomes from the current study showed that sleep facilitating factors are in a direct contrast to the sleep hygiene recommendations. Therefore, it is thus important for the sleep practitioners and healthcare providers to move beyond providing standardized sleep hygiene interventions. A Lifeworld led care model that pays attention to personal experiences, promotes sense of agency, evaluates both autism-specific strengths and struggles could and should complement biomedical approaches.

**Lay Summary:**

This is the first study to examine autistic adolescents’ self-reported sleep habits and factors which facilitate autistic adolescents’ sleep by employing adapted photo-elicitation interviews. This study is innovative in at least three ways. First, it examines the factors that may facilitate a good night’s sleep through personal accounts of autistic adolescents. Second, this is the first sleep study to adopt a collaborative, flexible approach to understanding positive sleep factors in the lives of autistic adolescents. This study employed a personalized approach into collecting, categorizing, coding, and analyzing qualitative data allowing autistic adolescents and the researcher to work together across key stages of data collection and data analysis. Third, we adopted a theoretical framework that allows us to consider autistic adolescents in both agency and vulnerability positions when it comes to their sleep difficulties. Our results highlight that sleep should be treated individually and in relation to the environmental and personal factors that affect each autistic person. Hence, researchers and professionals may benefit from working collaboratively with autistic adolescents with the aim to identify individual strengths and adopt a positive narrative around sleep. Furthermore, it is important to further examine both the daytime and evening factors that may affect bedtime and the quality and quantity of sleep as well as the role of intense focused interests and physical activities that cultivate positive feelings and help autistic people to relax before bedtime.

## Introduction

Adolescence is a significant period of development across different domains and includes significant reorganization in the brain (Feinberg and Campbell, 2010, pp. 56–65). Some of the rapid changes are seen in sleep patterns, elegantly named as “the perfect storm” when a combination of psychosocial and societal pressures negatively impact sleep quantity (Carskadon, 2011, pp. 637–647; Crowley et al., 2018, pp. 55–65). Adequate sleep has been associated with good health and high adoption of health-related behaviors (Chen et al., 2006, p. 59). Adolescent sleep issues appear in the media regularly proclaiming that parents and schools should ask their children to follow the sleep hygiene routine rules (Noland et al., 2009, pp. 224–230). At the same time, clinical and community awareness of the sleep issues of the typically developing adolescents appear to be increasing.

Autistic adolescents are particularly vulnerable to lifelong sleep problems, irrespective of their IQ (Richdale and Schreck, 2009, pp. 403–411). The most common sleep problems include sleep onset delay, nocturnal awakenings and shorter sleep duration (Malow et al., 2006, pp. 1,563–1,571; Miano et al., 2007, pp. 64–70; Giannotti et al., 2008, pp. 1,888–1,897). Several studies report that sleep disturbances have an impact on daytime functioning, including low mood and higher stress levels (Taylor et al., 2012, pp. 1,408–1,417; Hirata et al., 2016, pp. 86–99). Sleep initiation and maintenance have been associated as a risk factor for suicide attempts and, indeed, they increase the risk for suicide for autistic people across the lifespan (Hochard et al., 2020, pp. 1–10). Sleep research in the field of autism is growing rapidly, focusing on the examination of sleep patterns and their relationship with cognition and behavior (Richdale and Schreck, 2009, pp. 403–411; Sikora et al., 2012, pp. S83–S90; Cohen et al., 2018, pp. 391–403). Most of those studies primarily rely on parental questionnaires and some have used clinical objective sleep methodology. Despite the importance of sleep on many domains, we know very little about what helps autistic people to sleep and only from a few behavioral sleep interventions. Sleep education has been reported to be successful with younger children but has received much less study in adolescents. In fact, feasibility and acceptability of such methods are rarely reported by autistic adolescents themselves. The number of interventions that help autistic adolescents to sleep better is limited and there are only a few randomized control trials with the vast majoring focusing exclusively on behavioral therapy. Papadopoulos et al. (2019, pp. 341–350) identified 19 studies which included autistic children and adolescents but only 3 studies employed a randomized control trial methodology. However, findings of the three studies are mixed. For example, in the one study no effect of a standardized information pamphlet on insomnia compared to a control group (no intervention) was found.

The challenge in the field of sleep and mental health research in autism remains to establish research findings that address individual variations and autistic peoples’ perspectives of how they understand their sleep problems.

This is important for three reasons. First, most sleep studies with autistic population are based on parental reports about their children’s sleep (Reynolds et al., 2017, pp. 20–32). Consequently, routine history-taking only assesses pediatric sleep complaints that are problem-driven and developmentally focused (Moturi and Avis, 2010, pp. 24–37). Second, sleep research primarily relies on questionnaires and clinical objective sleep testing. While objective measures such as actigraphy, polysomnography, and videosomnography provide valuable information for both diagnostic purposes and treatment planning, they remain limited due to cost, availability, and feasibility to use with autistic people (Moore et al., 2017, p. 72). Last, objective measures of sleep may fail to capture the subjective sense of having a problem with sleep (Gregory and Sadeh, 2012, pp. 129–136).

A key issue is that it is simply not known how autistic children and young people choose to accommodate their sleep issues and what strategies they perceive as helpful. Accordingly, this study aims to explore autistic adolescents’ personal accounts of factors that contribute to a “*good night*” of sleep.

The increased recognition and reported prevalence of mental health conditions of autistic people across the lifespan along with the associated bidirectionality between sleep and psychopathology (Vandekerckhove and Wang, 2018, pp. 1–17) call for more research on sleep issues and sleep management. Understanding what helps autistic adolescents to have a “good night’s” sleep may have numerous benefits. For instance, early detection and sleep management can reduce the risk for ADHD (Owens, 2005, pp. 312–322; Knight and Dimitriou, 2019, pp. 423–436), depression (Lovato and Gradisar, 2014, pp. 521–529), and anxiety disorders (Willis and Gregory, 2015, pp. 125–131). Furthermore, a good sleep can lead to improvement of behavior which in turn, may have a positive impact on parental mental health (Wiggs and Stores, 2001, pp. 257–269). Finally, due to recent robust evidence that sleep is a strong predictor of quality of life (Deserno et al., 2019, pp. 794–801; Pavlopoulou and Dimitriou, 2019a, p. S296), identifying ways to improve autistic people’s sleep has become an urgent priority.

Therefore, examining personal sleep accounts of autistic adolescents with their active participation may help us further explore strategies to identify good sleep practices which will consequently have positive effects on their mental health and quality of life.

### Why Gather Personal Accounts From Autistic Adolescents

Qualitative research methods have theoretically played a minor role in sleep research, because sleep research primarily relied on questionnaires using predefined diagnostic categories when examining sleep habits and cognitions along with clinical objective sleep testing. The quantitative descriptors of sleep problems (e.g., sleep latency, sleep fragmentation etc.) have been traditionally favored while qualitative descriptions of the nature or experience of the sleep problems have been put aside. In addition, the use of qualitative participatory methods which are theoretically grounded in an experience-based approach may advance evidence base for addressing individual variations and autistic people’s perspectives to help the researchers understand their sleep issues and the consequences. The importance of collaborative mental health research is increasingly recognized, emphasizing the needs and preferences of adolescents and enabling them to contribute to their healthcare plans in their preferred ways (Gondek et al., 2016, pp. 325–343; Hindley and Whitaker, 2017, pp. 115–117).

The framework we present in the next section of this paper allows us to enhance our understanding of autistic people’s sleep through making their perceptions and evaluation of their sleep habits and sleep practices central and through acknowledging both autistic agency and autistic vulnerability. The latter is in line with recent calls for more research that advocates the ability of children and young people to act as dynamic research participants (Danby et al., 2011, pp. 74–84; Mandleco, 2013, pp. 78–82) and underlines their capacity to precisely share personal experiences (Pyle, 2013, pp. 1,544–1,558). This study welcomes this development as the exclusion of the autistic voice from the research can further reinforce the de-evaluation of autistic people.

### An Experience-Sensitive Approach Grounded in the Lifeworld of the Autistic Person

Evidence-based data on what matters to autistic individuals may help clinical practice to move away from the current dominant behavioral approaches and traditional sleep hygiene rules that were designed for the non-autistic population. Hence, the current study is guided by a humanizing value framework with a dedicated focus on qualitative research. When developing this framework, we were inspired by the existential-phenomenological tradition and an existential view of wellbeing which commits to viewing autistic people in both vulnerability and agency positions (Todres et al., 2009, pp. 68–77; Hemingway et al., 2012, pp. 26–27; Pavlopoulou and Dimitriou, 2019b, pp. 1–15). Lifeworld dimensions are described in Table 1 below.

**TABLE 1:** Dimensions of an adopted Lifeworld framework (Pavlopoulou and Dimitriou, 2019b).

Insiderness	Understanding autistic adolescents’ hopes, fears, struggles during every day routines and their personal and subjective views on sleep.
Agency	Promoting autistic adolescents’ abilities to take decisions with researchers, parents and clinicians around their involvement in sleep management.
Uniqueness	Autistic adolescents’ experiences are unique and should be seen as individuals with many identities such as female, student, friend, sibling, brother etc.
Sense making	Autistic adolescents’ interpretations of their school, community and families matter.
Personal journey	Researchers and practitioners should be proactive on facilitating each autistic adolescent’s personal journey planning and aspirations.
Sense of place	Autistic adolescents should feel welcome, safe across home, school and wider community. Chances to celebrate their identities and for them feel sensory comfort should be facilitated as a means to empowerment.
Embodiment	Each adolescent’s experience of living and being beyond a narrow definition of themselves based on deficits and disabilities. Enabling positive personal identities relevant to their strengths.
Togetherness	Autistic adolescents should have access to people they value and feel that they can share their worries/disagreements or ask for help, as well as to those who can share fun and intimate moments.

An experience-sensitive approach embedded in humanistic psychology could serve as a basis to explore sleep experiences with autistic people through the eight aforementioned dimensions. Their roots can be traced in ethics of care, compassion, and they declare a deep appreciation toward neurodiversity (Todres et al., 2009, pp. 68–77; Shakespeare, 2013; Milton, 2014, pp. 794–802).

## Materials and Methods

### Participants

Sixty participants were approached and 54 agreed to participate in the study. Ethical approval was obtained by UCL Institute of Education Research Ethics Committee, written informed consent was obtained from adolescents and their parent/guardian. A purposeful selection method was chosen in keeping with the requirements of a phenomenological tradition to have a sample with fairly homogenous experiences (Crouch and McKenzie, 2006, pp. 483–499). The inclusion criteria were as follows: (i) adolescents aged 12–17 years old with a formal diagnosis of autism; (ii) able to respond to a minimum of simple verbal, written or visual directions; (iv) being able to consent to participate in the photo-taking and interview process.

## Background Data Measures

Information on gender, age, diagnostic category, school placement, autistic features, and sensory profile were gathered to support the understanding of the background characteristics of the study’s sample and draw further conclusions about the qualitative results presented in this paper.

### Family, Medical and Educational History Questionnaire

The items included in this questionnaire were divided into three parts: (1) background information on their family’s demographics and socioeconomic status; (2) medical history including official diagnoses and use of any medication or involvement in therapy practice; (3) adolescents’ educational status.

### Childhood Autism Rating Scale 2nd Edition (CARS2)

CARS2 is a behavioral scale (Schopler et al., 2010) which measures behaviors including personal relations, imitation, emotional response, usage of body and objects, adjustment to change, and visual and listening response (Parkhurst and Kawa, 2018, pp. 1–3). This scale quantifies the presence of autistic features with each category being rated from 1 (age-appropriate behavior) to 4 (atypical behavior). Ratings on the CARS2 produce a total score ranging from 15 to 60, with scores of 15–30 corresponding to minimal-to-no autistic behaviors, 30–36 showing mild-to-moderate autistic behaviors, and 37–60 reflecting of greater manifestations of autistic behavior.

### Adolescent/Adult Sensory Profile Questionnaire (AASP)

The AASP is a 60-item self-report questionnaire that rates an individual’s (ages 11–65 years) reactions to everyday sensory events (Brown and Dunn, 2002). The questionnaire uses quadrant scores (Low Registration, Sensation Seeking, Sensory Sensitivity, and Sensation Avoiding) and each one comprises of fifteen questions. These include the categories of visual, auditory, touch, taste/smell, movement aspects, and a general category for activity level. Autistic adolescents rated themselves on a 5-point scale from 1 (almost never) to 5 (almost always). Scores were then summed to describe autistic adolescents’ sensory tendencies in each quadrant which were divided it into these categories: (a) as Much Less Than Most People, (b) Less Than Most People, (c) Similar to Most People, (d) More Than Most People, (e) or Much More Than Most People.

### Personalized Adapted Photo Elicitation Interviews

Each participant was asked to generate their own photos and determine their content by introducing subjects and ideas that are meaningful and important to them. This qualitative approach makes it possible to consider autistic adolescents as active participants in research and, to recognize their autonomy of thought, perspectives and ideas (Pavlopoulou and Dimitriou, 2020, 103556).

#### A Collaborative Approach: Involving Autistic Adolescents in Research and Knowledge Exchange

See Table 2 for an overview of the autistic adolescents’ involvement in the research phases.

**TABLE 2:** Overview of autistic adolescents’ involvement across different research phases.

Phase 1	Phase 2	Phase 3	Phase 4	Phase 5	Phase 6
Advisory research piloting	Recruitment	Participant-driven data collection	Participant-driven coding	Advisory group event preparation	Community event: open sleep day
Advisory Group (*N* = 6) Work included – Discussion of study objectives – Research design, procedures and tools – Establishing potential practices and audience for community engagement upon completion – Identifying themes for photo taking tasks – Piloting photo taking and adding set of verbal, non-verbal tasks to make interview more inclusive (visuals, objects)	Recruitment stage directly through charities, social media and local community support centers included – Obtaining informed consent from adolescents and their parents. – Training on ethics of photography.	Each adolescent was asked to take 10–15 photos and to keep notes or drawings for 1 week of: (1) The place they sleep and its surroundings. (2) Activities or objects that are related to their sleep habits. (3) Activities, objects, or people that show situations that are related to daytime and nighttime activities that help them to fall or stay asleep. (4) Objects, people, or drawings that show or are related to the bedtime thoughts and activities that help them to fall asleep. (5) Objects, drawings or actions that show how they feel or think about their sleep.	Individual interview meeting included: – Discussion of narrative using Showed protocol (Pavlopoulou and Dimitriou, 2020) to allow each participant to strengthen the message of their photos. What do you See happening here? Describe what the eye sees? What is actually Happening here? (What is the story behind this photo?) How does this relate to Our lives? Why does the problem/strength exist? How could this photo Educate others about the lives of young people? What can we Do about it? (What can researchers, parents, teachers and health professionals do in order to improve your life in the community?) – Codifying photos and personal narratives	– Lay down and select themes for exhibition based on emerging themes. Advisory group was asked to – Create ppt slides and collages with photos and post-it notes which capture key thematics for exhibition in Phase 6 based on data collected in Phase 5. – Generate two creative boards to identify stakeholders’ needs and preferred action points as part of an activity which will take part in Phase 6.	Community exhibition to share study results and identify post study action. Activities – Discussion panels with parents, 3rd sectors, school staff, developmental psychologists autistic adolescents and adults. – Two creative science workshops with participation of students from a local school. – A critical workshop for the researcher to discuss themes with community partners, participants’ families and other researchers. – Creative boards eliciting stakeholders’ needs and action points.

Prior to the home visit, each participant was reminded via email to upload the photos onto a tablet/iPad or print the photos to discuss them during the interview. Each home visit lasted from 60 to 90 min. Parents were only present on a few occasions upon request of the autistic participant.

### Process of Inductive Thematic Analysis

The process of data analysis is described in Table 3; based on (Sibeoni et al., 2017, p. 49; Sanders et al., 2019).

**TABLE 3:** Analysis stages.

Stage	Description	Rationale
1	*In situ* coproduced codes per case	Each autistic adolescent collected and reviewed their notes with the researcher during the 1–1 meeting. Each participant was asked after contextualizing their photos to create a personalized thematic map with photos and post-it notes with initial codes per participant. The researcher kept a copy along with her field notes and the explanations/comments/feelings offered by each autistic participant.
2	Synthesizing codes	The initial coproduced codes per case from stage 1 were further analyzed by grouping all codes together accompanied by sample key quotes and representative photos for each code. Each case’s codes were added to the ‘bank’ of codes that helped the researcher to start organizing meanings in patterns, describe meanings only in few words and photos
3	Identify initial themes	Selective transcription to obtain verbatim passages for use as evidence to match with codes created by autistic participants in stage 1 to compliment quotations, photos and notes from *in situ* analysis. Identifying patterns of meanings and organize them in preliminary themes. Add conceptual notes from previous stages to each theme. Reading themes and notes looking for commonalities and variations, comparing differences and similarities. All efforts are made for explicit naming of themes to match adolescents’ lived experiences. A lab researcher involved, 80% agreement achieved across random samples.
4	Finalize recurrent themes	Ensuring themes are summarizing the essence of conceptual notes gathered by autistic participants in stage 1. At this point, findings are written and rewritten using faithful descriptions while reaching a higher level of abstraction. Producing a coherent table of themes gathered into key domains of experience.
5	Seeking verification	The researcher returns the recurrent themes to a sub-sample of 20 adolescents to ask whether it captures their experience. 12 adolescents responded, 85% agreement achieved across all random samples.

## Results

### Background Characteristics of Participants

Fifty-four autistic adolescents aged between 12 and 17 years old (*M* = 14.6, *SD* = 1.7) were recruited through adverts on social media, emails to autism organizations and via autism charities websites. 32 identified as male, 20 identified as female and 2 identified as gender fluid. 51 had at least one brother or sister but none of them was sharing a bedroom with their siblings. All participants had a diagnosis of autism, 28 of them had an additional diagnosis of anxiety and/or ADHD, and/or dyspraxia and 18 of them were diagnosed with a mild or moderate learning disability. Thirty-six participants described themselves as White, 6 Asian, 7 Black and 5 other ethnic backgrounds. Household fell into socioeconomic group (NS-SEC) categories 1–3 using the employment information provided by mothers (Office for National Statistics, 2010). Thirty-two parents of the adolescents were married or in a domestic partnership, 12 were divorced, 4 were single and 6 preferred not to state.

Of the autistic adolescents participants, 36 were attending a mainstream secondary school, 14 were attending a special unit in mainstream secondary school or a special secondary school and 4 were home-schooled. Twelve participants were taking melatonin and 23 have used it in the past; 3 were taking medication for anxiety disorders and one was taking antidepressant medication for low mood.

Autistic adolescents’ CARS-2 scores ranged from 30 to 50 (*M* = 33.5, *SD* = 8.6). There were 42 participants who completed the sensory profile section of the questionnaire (Brown and Dunn, 2002). Low registration ranged from 25 to 73 (*M* = 45.5, *SD* = 8.3) which fell under the “much more than most people” category, scoring in the top 2% of the population. This means that many participants may have trouble reacting or low-intensity stimuli and might find it easier to focus on tasks of interest. Sensation seeking ranged from 21 to 56 (*M* = 39.7, *SD* = 9.3) indicating that most of the participants scored less than most people in the bottom 14% of the population. Sensory sensitivity ranged from 21 to. 56 with an average score (*M* = 46.07, *SD* = 4.2) of more than most people in the top 14% of the population, meaning that they often may need more structured input. Sensory avoiding ranged from 31 to 69 (*M* = 48.57, *SD* = 5.9) indicating that most participants scored more than most individuals in the top 14% of the population. That means that they may often benefit from less input available.

## Autistic Adolescents’ Personal Accounts

The following section offers a collection of illustrated verbatim and written extracts from the adapted photo-elicitation interviews. In presenting the extracts, minor changes were made to improve readability. For instance, minor hesitations, word repetitions and utterances have been mostly removed. All identifying information has been removed.

The themes and the master themes of the current study are described below in Table 4.

**TABLE 4:** Overview of master themes and subthemes.

Evening and bedtime factors that contribute to a good night of sleep	Personalized sleep habits- sleep autonomy. Parental company before bedtime – Different ways of being together may help to prepare for bedtime. Relaxation before closing my eyes.
Daytime factors that contribute a good night of sleep	The “feel good” daytime factor during school time. Physical activity helps. Spending Time on Favorite Hobbies and Activities.

### Master Theme 1: Evening/Bedtime Factors

Codes identified and coproduced with autistic adolescents can be seen in Table 5.

**TABLE 5:** Evening and bedtime factors sample of codes, quotes and photos.

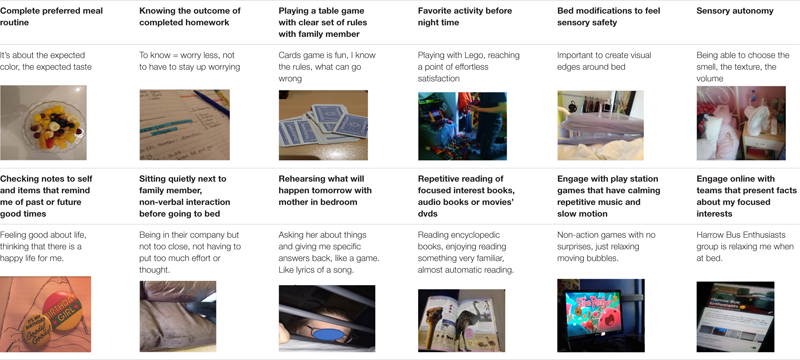

#### Personalized Sleep Habits During Bedtime

All participants (*N* = 54) felt that they were able to make the right decisions before and during bedtime. Most (*N* = 30) felt that their parents showed understanding and facilitated their need for sleep autonomy, even in cases when traditional sleep hygiene rules were not followed (e.g., avoiding screen time or games before bed). Autistic adolescents explained that they had personalized these general sleep rules to fit their need to achieve the right level of sensory stimulation and/or satisfaction from their engagement with these activities. All adolescents were able to develop independently varied ways of seeking sensory comfort before and during bedtime. A variety of different food, ranging from cold fruit and ice cubes to hot soups, helps them settle down. Similarly, preferences in nightwear ranged from light and short-sleeved/knee-high pajamas to heavy, fluffy pajamas fully covering their hands and legs.

I am sensitive during sleep time. Having a specific set of clothes helps me focus less on my skin sensations (…) it is what it is (laughs) so I can’t be bothered to change it and only adjusting summer or winter blankets makes it easier.

Many adolescents (*N* = 23) reported better sleep after removing labels from night clothing, and some (*N* = 16) enjoy applying a deep compression on the lower parts of their hands and upper parts of their legs. Others feel the need to hold a fluffy and soft item.

If I hold this [pointing to the photo of her holding a soft bunny toy] then I know where my hand is (…) My body is more comfortable.

Many participants (*N* = 27) described how a favorite smell from toys, clothes or oils or body creams may help them prepare to sleep, as illustrated in the quotes below.

We wash our clothes with a specific powder, I like this smell (…) I change my pajamas and wash them very often so that I can smell the smell of the laundry powder (…). It’s the smell of my sleep time.

I like the smell of my pillow (shows bottle of incense oil). I have 3 bottles like this.

Participants seemed to express a strong need and appreciation for a self-defined sense of comfort while in their beds. For example, many of them (*N* = 20) shared their need to visually define the edges of their bed using different items such as pillows, clothes, toys or bed rails.

I use different clothes to define visually the edges of my bed (…) I feel good to touch something concrete that reminds me this is the end of my bed…I know that there is a predefined limit for my body to move.

Also, many participants (*N* = 18) referred to the importance of blocking unwanted environmental noise in the room using different equipment with the fan being the most common in use.

#### Relaxation Before Closing My Eyes

The majority of the participants reported that listening to relaxing music and/or sounds/dialogs from familiar movies helped them to “*ease worries*,” “*sleep faster*,” or “*get into the sleep zone*” (*N* = 43).

These videos [on YouTube], they help me relax (…) stay calm in my room (…) focusing on the sounds, not really watching the video. Just listening to the sound to help me push away the worries about the next day.

I prefer my CDs ‘cause they don’t have adverts to interrupt (…) it’s like a blanket for all other noises from cars outside or neighbors (…) CDs with relaxing music or sometimes old movies that I have seen many times in the past (…) predicting the dialogs, remembering lines is like a game in my head, the sound of something familiar definitely helps me to sleep better.

I read my favorite book [pointing to an Encyclopedia with a focused interest on animals around the world] till at some point I feel sleepy and sleep (…) No other book, no because then I might need to put too much effort reading new words or thinking about the meaning of the words (…) This would actually make me worry.

Seventeen participants found popular self-help interventions helpful and generally described them as ‘*good tips*’ or ‘*wellbeing tips*’ that they learnt from a teacher or counseling therapist.

Writing a list about what’s going to happen the day after or plan A and plan B so that I feel prepared, I feel I have some sense of a plan.

I breath deep, it distracts me from my thoughts, I put an object, any object on my belly and watch my belly as it goes up and down while the air goes in through my nose and out through my belly.

Some participants (*N* = 14) have found several sleep routine rules suggested by a family member or a schoolteacher to be of help.

It was a few years ago when my mum suggested me a hot bath before going to bed (…) probably the most relaxing, you know, it’s you and the hot water and some bubbles.

Some participants (*N* = 10) tried meditation on phone applications and found them ‘*relaxing*,’ ‘*sometimes it stops my fears and just guides my thoughts into a calm place*’ and would use them a few times per week.

All adolescents reported benefits when parents facilitated their sleep autonomy and allowed them to engage in self-directed activities which involved high levels of repetition. Participants presented photos related to evening/before bedtime activities such as drawing, watching favorite animated characters cartoons, fantasy-themed objects such as role-playing cards, exercising favorite hobbies (gymnastics and playing music), playing video games and interacting online with others who share similarly focused interests. They all reported that such activities have a calming effect.

Dr. Who helps me sleep [points to his toy, a telephone booth from his Dr. Who toy collection] helps me to relax and forget all other thoughts in my head, then I can go bed bit easier.

It’s about getting to the right level (…) getting in my comfort zone, and thinking their characters, their weapons, their qualities (…) I like to memorize different details about the clothing, the faces and other stuff about the characters (…).

My whole room has a goth theme, I have all these little items that I like to collect too, which in many ways attract my interest. I find it very relaxing to explore their symmetry and patterns (…) their beauty stays with me and makes me feel good (…) it is a way to keep all worries away as your mind focuses on the beauty of these items (…) Trying to draw them as I lay down in bed is probably my most favorite way of switching off.

Many adolescents (*N* = 36) reported that they can relax better before and during bedtime on days when they do not have any extracurricular activities.

(…) Being home free to do what I want (…) choosing whether I want to talk or chat online or just keeping things to myself makes it easier (…) I go bed tired from things that I have enjoyed doing (…) feeling good at bedtime ‘This evening was not wasted, I did things I liked’ helps as I close my eyes.

#### Parental Company Before Bedtime –Different Ways of Being Together May Help to Prepare for Bedtime

This theme describes the perceived role of parents and the different ways in which their presence may act as a facilitator factor. Autistic adolescents’ personal accounts show that they identify three positive ways of being around their parents before bedtime: being next to them watching TV, playing a table game or another family game, or having a reassuring conversation as soon as they go to bed. They expressed the need to spend time next to their parents before going to their beds in the living room or the kitchen as this may give them a chance ‘*to wind down*’ and ‘*feel safe.*’

I like sitting next to my mother in the sofa (…) she is watching TV, we are silent but there is something about this silence that slows down my heartbeat and makes me feel very connected with her. We will not touch or talk or even move from our seat sometimes (…).

I like it when we all sit together to watch a film (…) I don’t have to worry about what I want to do or think too much (…) Few minutes after the movie begins I start watching and then will start to feel sleepy (…).

Some adolescents (*N* = 14) preferred spending time with their mothers playing cards and table games.

My mind gets busy with the game (…) the process is distracting me from other thoughts (…) which is a good thing I guess as I can then go to bed easier.

I like playing games, we play a game and I know when the game is done it is my time to go to bed.

Many teenagers (*N* = 19) stressed that parental company before bedtime gives them a chance to review the day in a gentle, compassionate way and make a plan for the day ahead.

My mum knows me, she knows how to ask me about things (…) she knows how to help me plan for things (…) I might go through a list of things with her (…) not so much to do things or to help me with things (…) it’s more like ‘I am here,’ ‘I am listening’ (…) rehearsing, like OK, I know more or less the order of what happens when or where tomorrow and then I will take it from there.

I don’t make lists cause I end up holding them and checking them for hours (…) When in bed, my mum will come and whisper all the things that are on my planner for the next day, like getting my PE kit, or to get my raincoat (…) it is like a list but not written (…) countdown till my birthday (…) or I might tell her what I read about the weather.

Some participants (*N* = 12) provide accounts of a more creative way of going through past or future events and tasks. They said that there is a tone of humor and dramatization in their dialogs with their mothers. This gives them a chance to acknowledge the successes and failures of the day while feeling ‘*it’s OK, no matter what happened*’ because ‘*mum reminds me a new day is ahead*’ and even when things did not turn out exactly as expected ‘*mum will find a way to make me laugh about it.*’ These participants valued the shared time as they ‘*shared feelings*’ and ‘*good vibes.*’

It is like a routine, she [mum] will come, touch the bed rail and sing to me (…) we do silly voices as we go through the things that will happen tomorrow or the things, we are saying silly stuff, like not really silly stuff, real stuff about homework and PE and other problems of the day in a way that is silly (…) makes me think that it does not matter so much after all (…).

Participants identified that their favorite bedtime conversations with family members do not have a specific communication purpose, have a playful tone, are characterized as a ‘good night’ ritual that promotes feelings of connection, are reciprocal, and allow the teen-mother dyad to enjoy a predictable and mutually fun time.

It is for us, for fun. I am in bed and mum will come stick her face next to me and we spend a couple of minutes holding the bed rail and repeating after my code words.

These conversations are distinct from other bedtime conversations as they have some musical qualities and mums are often expected to ‘*repeat words or respond in our private language*’ in which the adolescents say or complete phrases. These may include singing goodnight in different languages, improvising funny tales, or listing names of famous people, American presidents, members of the royal family and so on.

### Master Theme 2: Day Time Factors

Several codes were identified and coproduced by autistic adolescents and researchers. See Table 6 for a sample.

**TABLE 6:** Daytime factors sample of codes, quotes, and photos.

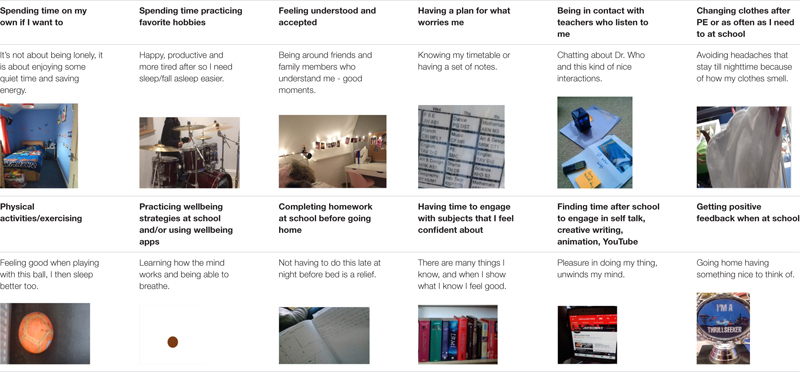

#### Spending Time on Favorite Hobbies and Activities

Autistic adolescents frequently captured their favorite activities during the daytime in their photographs. These included themed collections, self-talk activities, story writing, self-stimulatory body movement, drumming, and watching specific scenes from specific movies.

Moving my legs or hands, just following my body in a repetitive movement.

It [drumming] helps me to release all energy, I am only allowed to have a short session before bed, like 15 min or so (…) It feels relaxing after (…) I like the way my mind keeps a note of all of my moves, all the beats while I play (…) and when in bed it gives me something cool to think about.

Harry Potter (…) I don’t go for the whole movie, some parts that I like to watch over and over (…) The movement of heroes (…) the colors on a particular scene (…) fast forward to the answer to the mystery (…) sometimes I will watch only that scene (…) it excites me and calms me at the same time.

According to most autistic teenagers (*N* = 38), engagement with highly preferred items and activities helps them reach a state of calm happiness that cannot be compared with any other activity. This was often related to reaching a certain level of stimulation with activities that are not too easy or too hard, handling objects of great interest ‘*independently, without interruptions (…)* and developing a thinking routine around the ‘*details of the objects and its history.*’

They are all soft [the toy’s collection] and bring nice memories, some are presents from people I love (…) some are fidget toys to keep my hands busy and some (…) I have them all in a basket and go for them (…) [They] help me unwind my mind as I stare the ceiling trying to sleep.

For many adolescents (*N* = 20) a heightened sense of happiness, joy and calm was reported to result from successfully engaging in a favorite video game. Similar to the aforementioned activities, adolescents feel satisfied while gaming online, which in return makes it easier for them to fall asleep when they go to bed.

I am all smiley and happy, like some time ago when I constructed my own castle and a house for my dog in Minecraft (…) it is not real life, and that is the best thing about it. I forget the real life and get into a world that I am making my own stuff, and there are rules and everybody follows these rules (…).

I sometimes go online to chat a little bit as I play (…) It is about feeling less lonely, feeling confident to chat online about things I am really good at (…) and that is kinda important, finishing the day on a good note (…) like my voice is out there and some people around the world who have now read my lines.

#### The “Feel-Good” Daytime Factor During School Time

Most participants (*N* = 38) were able to identify key elements of their interactions and activities during and after school and how they relate them to a good night’s sleep. Eighteen participants reported that they sleep better when they know that they will enjoy the following school day. The factors contributing to a good day at school, cultivating positive thoughts and emotions during bedtime and facilitating a shorter sleep latency time include: attending to a focused interest subject; feeling cognitively able to respond to the lesson’s demands; having the chance to go through a lesson activity plan in advance; attending a club that they have set up; or having a good interpersonal connection with the subject teacher.

He will ask me how I feel, if it’s all good and how is my day (…) shows he cares about me and will talk about politics and answer my questions (…) feeling safe being around this teacher (…) falling asleep happy after checking his name on my timetable, something cool to look forward to.

Happy thinking in bed (…) knowing that I will speak to people I like at school helps me fall asleep (…) it is all about happy thinking sometimes (…) I think of a new joke to say to Mr. X during lunchtime as he is on duty every Wednesday. I might read a book with jokes to get ideas or create my own (…).

#### Physical Activity Helps

Participants (*N* = 38) linked activities such as gymnastics, biking, ball games, practicing musical instruments to a ‘*happier bedtime*,’ giving them a sense of hope as ‘*having fun during the day helps going to bed with less fear about the next day.*’

I like biking, I am good at biking (…) a sense of freedom that I don’t have during the other activities (…) a quiet space (…) action going up and down with my bike (…) hard to describe how I feel after, maybe more energized and less worried? Which is somehow related to the sleep question because I have noticed I sleep better on days I have enjoyed time on my bike.

I love playing with my basketball … not in a team, alone, shooting. I am very good at shooting. I love my ball. (…) because I relax and when I relax I get tired and get to sleep faster.

Swimming (…) I think I sleep faster on days I had a swim.

### Training and Engaging the Community

The momentum created at the individual level during each interview continued at the community level and involved researchers, local community and professionals working in schools, NHS and the 3rd sector. Delegates were invited to develop together action items based on the results of this study and conversations that took place on that day.

The community events were co-decided and co-organized with autistic adolescents. During the day of the event the researcher, the advisory group which included autistic adolescents who arrived with their parents on the day co-facilitated and/or attended activities which were co-designed with autistic adolescents. These included, among other things;

– A series of talks by sleep researchers and autistic service users covering sleep issues in neurodevelopmental conditions and syndromes and the ways these are related to learning, executive functions and mental health;
– Two interactive sleep workshops – where school students had the opportunity to learn why we sleep, how sleep works, how sleep is related to our physical and mental health;
– A collaborative poetry workshop about sleep and dreams;
– An opportunity to shape future sleep research using the suggestions board to help researchers understand what really matters to the autistic people’s sleep;
– Creative boards to facilitate the exchange of ideas and identification of community and research action points.

See Table 7 for an overview.

**TABLE 7:** Train and Engage overview of post study items.

Identified needs	Action item
Promoting participatory methodologies and qualitative designs which highlight acceptance of autistic variations and preferences over mainstream sleep hygiene rules	Sharing results with local mainstream schools through Photovoice exhibitions. Sharing results with autistic and non-autistic teenagers. Sharing results with parents’ charity groups. Writing up report for The John and Lorna Wing Foundation.
Promoting collaborative clinical formulations rooted in experience-based Lifeworld approach which recognize autistic people in both agency and vulnerability positions	Sharing results with CAMHS Tier 3 professionals across London. Sharing results with developmental and educational psychologists. Writing short blogs and reports for sleep charities who train nurses, occupational therapists and psychologists in sleep managements of autistic children and young people.
Promoting a positive narrative around autistic people’s sleep which recognizes autistic people’s ability to develop their personal sleep habits and practices.	Distribution of study report with links from media who covered the event. Posting study summary/key points on non-profit websites. Write up paper for a peer-reviewed journal. Webinars and podcasts for autism charities.
Raising awareness at a national and international level on the importance of flow, focused interests and the daytime “feel good” factor in autistic adolescents’ sleep.	Produce policy report with results of study for healthcare family practitioners and families. Use study data to increase funding via additional grants for non-profit autism sectors in 3 areas across United Kingdom. Incorporate a maximum of 10 photos and 1 multimedia video story approved by advisory group along with unlimited textual narratives when presenting in international conferences and research meetings. Short talks to Tier 3 and Tier 4 therapy teams.
Consider issues of intersectionality in autism and sleep research	Reinforce community-based participation within black and Asian communities by understanding which factors may facilitate their participation to sleep projects. Creating a network of service users, academics and community partners led by A 2nd Voice Charity. Consider methodologies to ensure the perspectives of those with minimum or no words are recognized and acted upon.
Explore the ways a Lifeworld-led approach may enrich community pediatric practice	Continuing Professional Development seminar talks to community pediatricians and nurses in London and Birmingham.

## Discussion

The current study highlighted the ability of autistic adolescents to evaluate and describe their sleep habits successfully using photo-elicitation approach. It is innovating in at least three ways;

(i) It aims to uncover which factors may facilitate a good night of sleep through personal accounts of autistic adolescents. This is significant because many autistic people suffer from sleep problems which affect their cognitive and mental health. Sleep assessments typically include parental reports on behavior, sleep diaries and objective sleep measures such as actigraphy. We suggest that using visual ways to elicit sleep experiences of sleep may complement the well-established methods. The involvement of autistic children and adolescents is crucial and ensures that innovations in practice draw on work done in collaboration *with* autistic people and their families, rather than taking the detrimental approach of making assumptions *about* them.
(ii) To adopt a Lifeworld framework that accounts for individual differences in understanding positive sleep factors in the lives of autistic adolescents and support the investigation of personalized sleep hygiene practices in this population. Also, knowledge about autistic adolescents that is derived directly from fieldwork experience co-produced with autistic adolescents may facilitate the development and co-delivery of quality services, programs, and policies.
(iii) To report the importance of agency and focused interests of autistic adolescents, elements that have remained poorly recognized and insufficiently researched in the current cognitive and behavioral approaches in sleep management.

The personal accounts of autistic adolescents in our study verify the importance of sensory comfort and lowering anxiety before and during bedtime and are in line with the work of Mazurek and Petroski (2015, pp. 270–279) who found strong links among sleep problems, sensory responsivity and anxiety.

Furthermore, our results show that adolescents have a wide range of personalized sleep habits which help them to accommodate a good night’s sleep. For example, they feel that predictable interactions with family members may facilitate a relaxing routine before bedtime while daytime factors such as physical activity, positive school experiences and sufficient time for hobbies will also result in good nocturnal sleep.

The results emphasize the humanistic value and holistic contextuality of lived experience in sleep research. Our results suggest that it is imperative to facilitate sleep autonomy during bedtime and shift from generalized sleep hygiene practices created and used by non-autistic people to personalized sleep practices for the autistic population.

Given the importance attached to the evidence-based practice in NHS (Department of Health, 2007) it is important to consider the preliminary data this study offers towards a holistic approach in treating autistic adolescents’ sleep issues in relation to the environmental and personal factors that affect each autistic person individually. Our study argues for a new emphasis on evidence-based practice with the contributions of service users that honors their everyday experiences of sleep. In line with the work of Glasby and Beresford (2006, pp. 268–284), we suggest a range of steps and materials which can be used to not only collect their views but also involve them in the research process of sleep studies. To achieve that, we suggest a Lifeworld framework, rooted in lived experiences which allows for the distinct features of autistic adolescents’ habits and practices to be in the center of future research and practice. Furthermore, an inductive participatory process that relies on an experienced based approach may allow new necessary knowledge to be co-created with autistic adolescents for this under-researched area in the sleep research in autism. Finally, it is recommended that the impact of daytime stress and the ways it may affect sleep, as experienced by autistic people themselves, should be further investigated.

### Implications for Practice

Given the heterogeneity of the autistic population and the shortage of mental health providers for the autistic population, it is very important to develop individualized sleep support that is meaningful to autistic adolescents. Meaningful involvement demands a personalized approach, empowering adolescents to understand and contribute to their mental health (Bee et al., 2016, p. 56). In this study, based on the autistic adolescents’ personal accounts shared in the results section we have identified 4 key domains who interact with each other and together they have a positive impact on sleep which is discussed below.

#### Focused Interests

Activities such as focusing on special interest objects may help autistic people to achieve a flow state (Csikszentmihalyi and Csikzentmihaly, 1991). This type of activity might be interpreted as a meaningless or repetitive behavior by parents and can be interrupted to redirect the autistic person to comply with a sleep hygiene rule. Thus, parents and professionals should consider that focused interest activities, objects, and thoughts might help the autistic person to be in a flow state which is beneficial for reducing stress. Promoting and planning daily activities that incorporate focused interests can be an important area in future sleep management practice. Identifying flow states for autistic people and trying to develop a flow plan during daytime and before bedtime might have a positive impact on sleep.

#### Physical Activity

For autistic adolescents, it is important to have opportunities to exercise in their own time and space to avoid experiences which can interfere with their personal space, sensory issues, anxiety-provoking group interactions or body confidence. Exercise has been shown to reduce and stabilize cortisol levels over time (McDonnell et al., 2015, pp. 311–322) which can have a positive effect on sleep.

#### Sense of Agency During Daytime

We found potential evidence on the importance of the daytime ‘feel good’ factor as defined by autistic adolescents on sleep. Being able to have more control and choice on how to spend time at school and home may cultivate positive feelings that promote better sleep. Based on the current findings, autistic adolescents are most likely to have a good night’s sleep when they interact with people who meet their needs. The subjective experience and the concept of autistic adolescents’ locus of evaluation should be prioritized if we aim to understand the social determinants of wellbeing. It is thus important for sleep therapists to empower adolescents and their families to evaluate the impact of daily activities and develop a balance between obligatory and desirable activities.

#### Sensory Autonomy During Bedtime

It has been well established that autistic people experience unique experiences and a sense of control over sensory stimuli may have an impact (Robertson and David, 2015, pp. 569–586). The autistic adolescent should be seen as the expert of his/her sensory profile and allowed to modify his/her environment. Hence, practitioners need to consider that the parental as well as their own perspective may promote or hinder sleep activity. Sleep should be treated at an individual level in relation to the environmental factors: flexibility, agency and personalization may prove more helpful than manualized approaches that do not consider the autistic perspective. More comprehensive sensory-based assessment through focused conversations with adolescents and their family members may advance the development of sleep management by identifying factors that influence the levels of sensory stress experienced by autistic people before and during bedtime.

### Limitations

The findings of the study should be considered in light of several limitations. Primarily, the methodological approach did not allow autistic adolescents with severe learning disabilities and/or minimum or no use of spoken words to share their sleep habits and experiences. It is difficult to determine whether our results are representative of autistic adolescents with additional co-occurring conditions such as epilepsy. However, I argue that it is important for sleep researchers and service providers to work actively toward eliciting views from autistic people of all abilities and communication styles.

Despite the methodological innovation to include some steps toward engaging autistic adolescents into collecting and analyzing their own data as well as preparing material in order to train and engage the public, further steps need to be taken for a more fully developed co-production model. In the future, such efforts should focus on combining the knowledge, skills and experience of autistic adolescents who use, deliver and commission services to further promote positive change and improve lives and outcomes.

## Conclusion

Our study provides evidence that healthcare practitioners should go beyond providing a standardized sleep hygiene handout and instead collaborate with autistic adolescents to co-create a personalized sleep set of habits. These may include the development of daytime activities which increase predictability, flexibility to achieve sensory comfort and activities, relationships and objects which promote a sense of worth. Research and practice that focus on the qualitative descriptions of sleep by adopting a Lifeworld approach (Pavlopoulou and Dimitriou, 2019a, p. S296), may further enhance the development of an evidence base for engaging children and young people in health and care by using their strengths and what works for them, irrespective of mainstream sleep hygiene rules. Consequently, we will be able promote good practices that help healthcare practitioners to attune their intervention plans to the autistic adolescents’ preferences.

## Data Availability Statement

The datasets generated for this study are not readily available because they include sensitive data (photos of teens houses, family members, personal objects, and school timetables). Requests to access the datasets should be directed to corresponding author.

## Ethics Statement

The studies involving human participants were reviewed and approved by UCL IOE Ethics Committee. Written informed consent to participate in this study was provided by the participants’ legal guardian/next of kin.

## Author Contributions

GP co-designed the study with adolescents, coordinated all phases, and contributed to all sections of the final manuscript.

## Conflict of Interest

The author declares that the research was conducted in the absence of any commercial or financial relationships that could be construed as a potential conflict of interest.
