# Corrigendum: A Good Night's Sleep: Learning About Sleep From Autistic Adolescents' Personal Accounts

**DOI:** 10.3389/fpsyg.2021.657385

**Published:** 2021-04-07

**Authors:** Georgia Pavlopoulou

**Affiliations:** ^1^Department of Psychology and Human Development, Institute of Education, University College London, London, United Kingdom; ^2^Anna Freud National Centre for Children and Families, London, United Kingdom

**Keywords:** sleep, autism (ASD), phenomenology and care ethics, adolescence, mental health – related quality of life, autistic wellbeing

In the original article, Dr. Dimitriou was mentioned in the Acknowledgments section. Dr. Dimitriou has requested that their name be removed from this section.

The corrected Acknowledgments section is shown below.

## Acknowledgments

The author would like to express her gratitude to Prof. Richard Mills who worked with the author to secure funding for this work. With gratitude to Jon Adams, Theodora Kokosi, Tatiana Souteiro Dias, Mark Devonport, Vanessa Bobb, George-Philip Kantianis, Helen Craig, Cos Michael, and Maria Kambouri who have been providing expertise and supportive comments which greatly improved the participatory research steps and knowledge exchange activities in order to maximize the impact of this work in the community. The author would like to thank London Autism Charity, Parenting Special Children Charity, A 2nd Voice Charity and Resources for Autism Birmingham branch for supporting her during the recruitment process. Last but not least, the author would also like to express her gratitude to the adolescents for sharing their pearls of wisdom with her during the course of this research.

The authors apologize for this error and state that this does not change the scientific conclusions of the article in any way. The original article has been updated.

